# Sanitas

**Published:** 1886-01

**Authors:** E. S. Talbot

**Affiliations:** Chicago, Ill.


### SANITAS.

BY E. S. TALBOT, M. D., D. D. S., CHICAGO, ILL.

My opinion of the above liquid having been sought personally and by letter, I consider it best to publicly express it, as others may be interested. I was induced to try its disinfecting qualities from the following remarks of Dr. Harlan, quoted from page 269, *Ohio State Journal* for June: "Sanitas is a disinfectant. * * * Its elements are capable of combining with sulphuretted hydrogen gas and other products of decomposition, and thereby destroying or removing them." The first quality manifested was its odor, which remained for a great length of time. I made two applications to the root of an incisor, and was unable to treat further, as the patient could not endure the taste in the mouth. I passed a probe into the canal, and the odor of Sanitas was stronger than sulphuretted hydrogen. I suspected that it had not disinfected, but its own powerful odor had covered other odors. I consequently commenced a series of experiments, and think any one may confirm the results if he will do likewise.

I put a small quantity of $H_2S$ (Sulphuretted Hydrogen) in a test tube and added some water, then put a drop of acetate of lead on a strip of white paper and inserted it into the tube, which immediately turned dark. I put a few drops of Sanitas into the test tube, corked and shook it well, and let it remain a few minutes. I moistened the other end of the white paper with acetate of lead, and dipped it into the test tube. The dark color is the same as before, showing that no chemical change occurred by adding Sanitas. There is a little $H_2O_2$ (Peroxide of Hydrogen) in Sanitas, but it is so stable that it does not unite with the $H_2S$, and I therefore see no reason why we should keep such an odorous liquid about the office, merely to get $H_2O_2$ when the same can be procured in full strength, and is a much pleasanter remedy to use. Also upon page 260 of the INDEPENDENT PRACTITIONER, we find "a great many who camplain of want of success with Sanitas can only blame their own carelessness." This statement implies that the writer has a knowledge of the article in question, and that those who have been unsuccessful in its use have not the article to blame, but themselves alone. I differ, most decidedly, with Dr. Harlan on this point, and feel that had there been a more thorough understanding of the properties of Sanitas, it would not have been recommended to the profession as a disinfectant.

